# A Potential Method for Rapid Screening of Amphioxus Founder Harboring Germline Mutation and Transgene

**DOI:** 10.3389/fcell.2021.702290

**Published:** 2021-08-12

**Authors:** Jiaqi Zou, Xiaotong Wu, Chenggang Shi, Yanhong Zhong, Lei Zhang, Qiuning Yan, Liuru Su, Guang Li

**Affiliations:** State Key Laboratory of Cellular Stress Biology, School of Life Sciences, Xiamen University, Xiamen, China

**Keywords:** amphioxus, mutation, transgene, tail tissue, gametes, correlation

## Abstract

Amphioxus is a promising model organism for understanding the origin and evolution of vertebrates due to its basal phylogenetic position among chordates. We here compared the mutation efficacy and mutation type of tail tips and gametes of amphioxus founders injected with Cas9 protein and six different sgRNAs targeting five distinct genes, and revealed a strong correlation for mutation efficacy and a mild correlation for mutation type among the two tissues. In addition, we also observed a positive relationship between gene insertions observed in tail tips and gametes of amphioxus founders injected with Tol2 transposase and two different transgenic constructs. Finally, we showed that amphioxus larvae which had their tail tips cut at the 3–4 gill-slit stage were able to recover within 6 days and developed a normal number of gonads at the adult stage, and that F0 larvae carry similar mutation efficacy and type in the posterior end to that in the tail tips after their metamorphosis. Together, these findings suggest a great potential for obtaining valid amphioxus founders with desired mutations and transgenes at as early as the early larval stage, which will certainly speed up the generation of amphioxus mutants and transgenes and make it more cost- and labor-effective.

## Introduction

The phylum Chordata constitutes of three subphyla: Cephalochordata, Urochordata and Vertebrata. Among them, Cephalochordata is the most basal subphylum (Delsuc et al., 2006) which comprises around thirty living species, commonly known as lancelets or amphioxus. Anatomically, amphioxus have segmented muscles, a dorsal hollow neural tube, notochord, gill slits, a through gut and postanal tail while lacking bones, appendages, paired sensory organs (such as eyes and ears), an adaptive immune system, and a well-developed brain and visceral organs. Developmentally, amphioxus embryos are similar to vertebrate embryos in the sense that they undergo blastulation, gastrulation and neurulation. Nevertheless, they are like a single-cell layered ball at the blastula stage, gastrulate simply by invagination of the vegetal pole toward the animal pole, and do not form neural crest cells and definitive placode at the neurula stage. Genetically, the amphioxus genome has retained many ancestral chordate traits but has not undergone massive duplications like that in vertebrates (Putnam et al., 2008). Due to the above-mentioned advantages, amphioxus has been considered as a promising model organism for studying the origin of vertebrates and the evolution of chordates since they were discovered in the 1,800s (Bertrand and Escriva, 2011).

The thirty described amphioxus species are divided into three genera: *Branchiostoma*, *Asymmetron*, and *Epigonichthys*, and most of them belong to genus *Branchiostoma* (Poss and Boschung, 1996). Up to date, the genomes of three species of *Branchiostoma*, *B*. *floridae*, *B*. *belcheri*, and *B*. *lanceolatum*, have been sequenced (Putnam et al., 2008; Huang et al., 2014; Marletaz et al., 2018). In addition, methods for year-round spawning induction has been developed for all three species (Benito-Gutierrez et al., 2013; Li et al., 2014b, 2017), and TALEN-based genome editing and Tol2 transposase-mediated transgenic methods have been introduced in *B*. *floridae* and *B*. *belcheri* (Li et al., 2014a, 2017; Shi et al., 2018). Very recently, the CRIPSR/Cas9 system has also been applied for genome editing in *B*. *floridae* (Su et al., 2020). These advances have greatly accelerated the rate of establishing amphioxus as a model organism, and enables researchers to robustly dissect the function of amphioxus genes (Hu et al., 2017; Li et al., 2017; Ren et al., 2020; Zhong et al., 2020; Zhu et al., 2020). However, current methods used for identifying valid founder amphioxus (F0) carrying desired mutations or transgene are time-consuming (Holland and Li, 2021) since they rely on animal spawning that normally takes at least 3 months (from their births) for *B*. *floridae* or more for other amphioxus species (Zhang et al., 2007; Escriva, 2018). Moreover, to ensure success, especially for transgene and mutation sites of low efficacy, more than several dozen F0 animals need to be raised. This would take more effort and slow down the growth rate of the founders due to high culture density. To overcome this drawback, we here compared the mutation efficacy and mutation type induced by Cas9/sgRNA ribonucleoprotein (RNP) among tail tips and gametes of F0 individuals, and found that both of the two parameters are well correlated between the two tissues. We also observed a positive relationship of successful transgene insertion between tail tips and gametes of F0 individuals injected with Tol2 transposase and two vectors. Finally, we demonstrate that amphioxus larvae whose tail tips were cut at 3 gill-slit stage were able to recover within 6 days and develop a normal number of gonads at the adult stage, and that F0 larvae carry similar mutation efficacy and type in the posterior end to that in the tail tips after their metamorphosis. These results together indicate that it is possible to identify F0 amphioxus carrying desired mutations or transgenes at as early as the 3 gill-slit stage. This advantage will greatly accelerate the generation of genetically modified amphioxus animals.

## Materials and Methods

### Animal and Embryo Cultivation

Amphioxus *Branchiostoma floridae* were obtained from Dr. Jr-Kai Yu’s laboratory at Institute of Cellular and Organismic Biology, Academia Sinica, Taiwan. They were maintained and induced to spawn following the protocols as we described before for *B*. *belcheri* animals (Li et al., 2012, 2014b). Fertilization and embryo cultivation were carried out as previously reported unless otherwise stated (Liu et al., 2013).

### Cultivation of Amputated Larvae

The posterior end of amphioxus larvae (with 3–4 gill slits) were amputated with a double-edged blade under a stereoscope. They were then raised in a petri dish (diameter = 6 cm) containing filtered sea water placed in a 30°C incubator and fed with *Dicrateria zhangjiangensis* twice a day. Sea water was changed once per day. After metamorphosis, they were moved to a 5 L plastic barrel and raised as previously described (Li et al., 2012, 2014b). Photographs were taken with an inverted microscope (Olympus, IX71) or an SZX10 fluorescent stereoscope (Olympus, Japan).

### Mutant and Transgenic Founder Generation

We used the CRISPR/Cas9 or Tol2 system reported previously to generate mutant or transgenic founders (Shi et al., 2018; Su et al., 2020). Seven sgRNAs, which target *Invs* (one sgRNA), *Wnt3* (one sgRNA), *VegT* (one sgRNA), *Mop* (one sgRNA), *Cyp19a2* (two sgRNAs), and *Tesd* (one sgRNA) genes, respectively, and two *mCherry* transgenic constructs, that, respectively, include the promoter (1,784 bp) of the *Xenopus laevis Slug* gene (Vallin et al., 2001) and that (3,285 bp) of the *B*. *floridae Pou4* gene, were used. gRNA targeting sequences and primers used for transgenic vector construction were, respectively, listed in Supplementary Tables 1, 2.

### Mutation Efficacy and Mutation Type Detection in Tail Tips and Gametes of F0 Amphioxus

F0 founders were crossed with wild type amphioxus to generate F1 embryos. For mutation efficacy detection, the tail tip of F0 amphioxus, semen from male mutants and F1 gastrula embryos (around 50) of female founders were lysed to get genomic DNA using the Animal Tissue Direct PCR kit (FOREGENE, Chengdu, China). PCR amplification and restriction enzyme digestion assay was performed as previously described (Li et al., 2017). Mutation efficacy was estimated by comparing band intensity between uncut and all bands [uncut/(uncut + cut)]. Band intensity was quantified using software implemented in the Tanon Gis system (Tanon, Shanghai, China) (Su et al., 2020). For mutation type detection, the PCR products obtained above were further cloned into the pGEM-T easy vector (Promega, United States) (except *Tesd* gene, whose PCR products were ligated after digestion with *Eae*I). Clones with mutated target site were identified as described above using restriction enzyme digestion assay, and then sequenced to determine their mutation types. Primers and restriction enzymes used for each gRNA were, respectively, shown in Supplementary Tables 1, 3.

### Genotyping of Transgenic Amphioxus

Genomic DNA of tail tips and gametes of transgenic F0 founders was obtained as described above, and used as the template to amplify a 624 bp fragment of pMini-mCherry backbone with primers mCherry-F1 (5′-CTTCGCCTGGGACATCCTGT-3′) and mCherry-R1 (5′-GCATTCTAGTTGTGGTTTGTC-3′) under the following conditions: 95°C for 5 min, 38 × (95°C for 30 s, 62°C for 20 s, 72°C for 30 s), 72°C for 5 min, and hold at 4°C. Ten microliters of each PCR product was used for gel electrophoresis and those including the 624 bp band indicated successful insertion of the target sequence.

### Statistical Analysis

Data were analyzed using a two-tailed unpaired *t*-test, correlation or simple linear regression with GraphPad Prism 8.3.0. We set gamete efficacy as x value and tail efficacy as y value for correlation analysis of mutation efficacy. And for correlation analysis of mutation type, in each individual, we regard the proportion of one gamete mutation type as x value and the same type of tail tip as y value.

## Results and Discussion

### The Mutation Efficacy Measured in Tail Tips of F0 Amphioxus Correlates Strongly With That in Their Gametes

**FIGURE 1:**
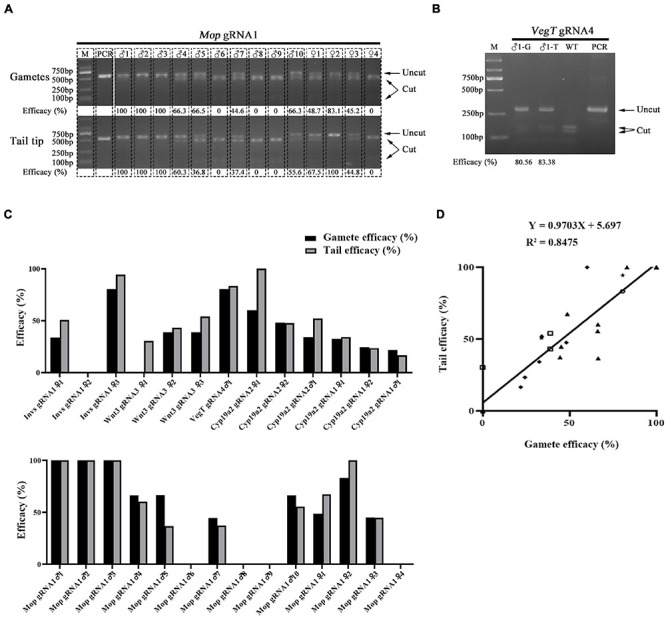
Correlation of mutation efficacy between tail tips and gametes of F0 individuals. **(A,B)** Mutation detection in tail tips (T) and gametes (G) of F0 individuals injected with *Mop* gRNA1 **(A)** and *VegT* gRNA4 **(B)**. The induced mutation efficacies (estimated as percentages of uncut PCR products) are labeled under the gel image. **(C)** Summary of mutation efficacies detected in tail tips and gametes of all F0 individuals examined. **(D)** Correlation analysis of efficacies between tail tips and gametes of F0 individuals. Efficacy of each gRNA is marked with a different symbol. The linear equation is *Y* = 0.9703X + 5.697, *R*^2^ = 0.8475.

Both male and female amphioxus have more than twenty pairs of gonads, which are distributed along the ventral tip of the myoseptal walls in the middle part of the body. Staining of marker genes suggests that amphioxus primordial germ cells (PGCs), from which the gonad cells derive, are located within the tail bud and proliferate together with the tail bud cells from the mid-neurula (8 pairs of somites) stage (Zhang et al., 2013). During posterior somite formation from the tail bud, some of these PGCs are thought to be deposited near the forming myomere boundaries and then settle at the ventral tip of the myoseptal walls during subsequent development (Zhang et al., 2013). This finding shows a close relationship between tail and gonad tissue during amphioxus development, and raises a possibility that gonads of F0 animals injected with RNP would carry similar mutation efficacy compared to tail tissue. To test this, we examined the mutation efficacy of tail and gonad tissue of F0 individuals injected with Cas9 protein and gRNAs targeting *Invs*, *Wnt3*, *VegT*, *Cyp19a2*, and *Mop* genes. This includes six gRNAs in which two targeting the *Cyp19a2* gene and one targeting each of the other four genes. After being injected (gRNAs for each gene were injected separately), the embryos were raised to adulthood and their tail tips and gametes (for females their offspring generated by crossing them with wild type males were used) were then collected for mutation efficacy detection using a restriction endonuclease assay. In total, three females for *Invs* gRNA1 and *Wnt3* gRNA3, one male for *VegT* gRNA4, two females and one male for *Cyp19a2* gRNA1 and 2, and four females and ten males for *Mop* gRNA1 were analyzed. Among these 24 individuals, five (*Invs* gRNA1 ♀2, *Mop* gRNA1 ♂6, ♂8, ♂9, and ♀4) carried no mutation in either tails or gonads, one showed mutations (30.46% efficacy) in tail tissue but not in gametes, and the remaining 19 exhibited mutations (16.67–100% efficacy) in both tails and gametes (Figures 1A–C and Supplementary Figure 1). Correlation analysis revealed that the mutation efficacies detected in the gametes of these animals were highly correlated with those detected in their tail tips (*Y* = 0.9703X + 5.697, *R*^2^ = 0.8475) (Figure 1D). This result indicates that the germline mutation efficacy of F0 amphioxus can be well indicated by the mutation efficacy of their tail tips.

### Correlation of Mutation Type Between Amphioxus Tail Tips and Gametes

**FIGURE 2:**
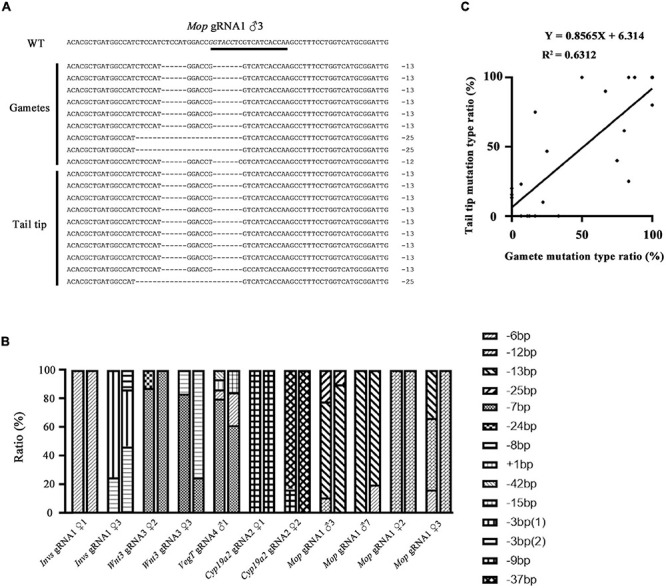
Correlation of mutation types between tail tips and gametes of F0 individuals. **(A)** Mutation types detected in tail tip and gametes of a F0 male (♂3) injected with *Mop* gRNA1. The gRNA target site is underlined and the restriction enzyme recognition sequences is marked in italics. **(B)** Summary of mutation types detected in gametes and tails of all F0 individuals. For each individual, the first column represents mutation types detected in the gametes and the second column represents those detected in the tail tips. Each type of mutation is marked with a different symbol. **(C)** A linear correlation analysis on the percentage of each mutation type between tails and gametes. For those with multiple mutation types, each type was treated as an independent dataset. The linear equation is *Y* = 0.8565X + 6.314, *R*^2^ = 0.6312.

We then compared the mutation types between the gametes and tail tips of F0 amphioxus to see if they are also correlated. Eleven individuals used in the analysis above were selected. Figure 2A showed an example of the mutation types identified in a male injected with *Mop* gRNA1 (*Mop* gRNA1 ♂3). There were, respectively, three and two types of mutations in the gametes and tail tip. Remarkably, two of these mutations (−13 and −25 bp) were shared by both the gametes and tail tip, and the 13 bp deletion was a major type of mutation in both tissues (6/9 = 66.6% in gametes and 9/10 = 90.0% in tail). The −12 bp mutation found in the gametes was not detected in the tail tip, which might be caused by limited number of clones we analyzed. Similar results were also observed in the other ten animals (Figure 2B and Supplementary Figure 2). In some extreme cases (*Invs* gRNA1 ♀1, *Mop* gRNA1 ♀2, and *Cyp19a2* gRNA1 ♀1), an identical mutation type was detected in both gametes and tail of the animals. We then conducted a linear correlation analysis on the percentage of each mutation type between the two tissues of the animals examined (for those with multiple mutation types, each type was treated as an independent dataset). The result showed that the mutation types present in the gametes and tails were well correlated and unified into a linear equation (*Y* = 0.8565X + 6.314, *R*^2^ = 0.6312) (Figure 2C). From these, we infer that the mutation types, at least the major one, in the gametes of F0 amphioxus could be predicted by the mutation types present in their tail tips.

### Prediction of Germline Transmission of Transgene in F0 Amphioxus by Analyzing Their Tail Tips

**FIGURE 3:**
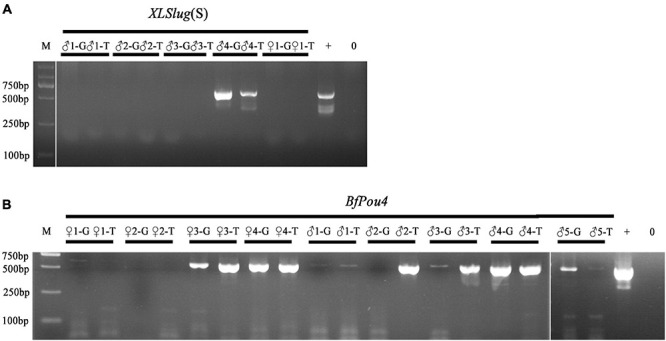
Detection of transgene in tail tips and gametes of F0 animals. **(A)** Analysis of F0 animals injected with *XLSlug*(S)-*mCherry* construct. **(B)** Analysis of F0 animals injected with *BfPou4*-*mCherry* construct. The presence of the 624 bp band indicates successful insertion of target sequence. + means positive control and − means negative control.

The Tol2 transposase-mediated transgenic method has been recently introduced into *B. floridae* (Shi et al., 2018). Screening of F0 founders carrying the desired transgene is currently conducted after the founder animal spawns, by detecting the presence of transgene in their gametes or offspring embryos using PCR method (Holland and Li, 2021). To see if germline transmission of transgene in F0 amphioxus can be predicted by the presence of the transgene in their tail tissue, we conducted PCR analysis on both tissues of 14 individuals injected with *Tol2* mRNA and *XlSlug*(S)-*mCherry* (5 animals) or *BfPou4*-*mCherry* (9 animals) constructs. The result showed that amongst these animals, seven (*XlSlug*(S) ♂4, *BfPou4* ♀3, ♀4, ♂1, ♂3, ♂4, and ♂5) contained the transgene in both gametes and tail tips, one (*BfPou4* ♂2) in only the tail but not gametes, while we failed to detect the transgene in the remaining six [*XlSlug*(S) ♂1, ♂2, ♂3, ♀1, and *BfPou4* ♀1 and ♀2] in either gametes or tail tissue (Figure 3). Since we could not directly determine transgenic efficacy in the tail tip, we did not know if it is well correlated with that in the gametes. However, the above analysis suggests a strong correlation in the presence or absence of a transgene between the gametes and tail tips of F0 amphioxus. This demonstrates that the germline transmission of transgene in F0 amphioxus could be reliably predicted by detecting the transgene in their tail tips.

### Amphioxus Larvae With the Posterior End Removed Are Able to Survive to Adulthood and Grow a Normal Number of Gonads

**FIGURE 4:**
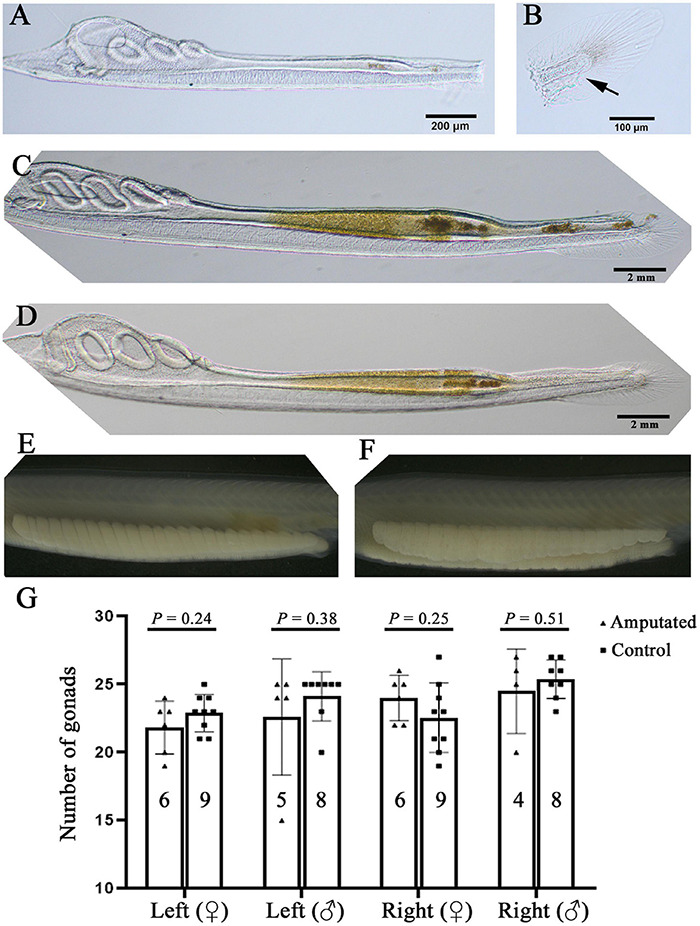
Amphioxus larvae that have undergone posterior end amputation are able to regenerate and develop a normal number of gonads. **(A)** A representative larva just after posterior end amputation. **(B)** An example of the amputated posterior end. Arrow marks the anus. **(C)** An example of larva 3 days after posterior end amputation. Note that its posterior end was almost regenerated. **(D)** A larva with fully recovered posterior end 6 days after amputation. Left-side gonads of a male **(E)** and a female **(F)** whose posterior end was amputated at the larval stage. **(G)** Statistical analysis of gonad number in amputated and wild type (control) animals. Males and females, and their left-side and right-side gonads are compared separately. Animal numbers examined are shown in the columns. *P*-values calculated using a two-tailed unpaired *t*-test are shown above the columns.

The results shown above demonstrate that F0 amphioxus have a similar mutation or transgene insertion rate between their gametes and tail tissue, echoing the close relationship between PGCs and tail bud in early development. This finding indicates a possibility to identify F0 amphioxus carrying valid germline mutations and transgenes at early stages before sexual maturity by assaying gene editing efficiency using tail tissue. As an initial step to test the above possibility, we amputated the posterior ends of 14 amphioxus larvae with 3–4 gill slits which include the tail bud and most of the tail fins (slightly anterior to the anus) (Figures 4A,B and Supplementary Figure 3A) which contained enough cells for genotyping. We found that most of these larvae could recover partially 3 days after amputation, and regenerate the entire posterior end (including the tail bud, anus and tail fin) 6 days after amputation. This demonstrates that amphioxus larvae have strong posterior end regeneration potential and are able to recover after amputation at a much faster rate than adults (Somorjai et al., 2012; Figures 4C,D and Supplementary Figures 3B,C). Eleven of the 14 amputated larvae survived to adulthood. Notably, they all grew a normal number of gonads on either side, similar to the untreated wild type animals (Figures 4E–G and Supplementary Figure 3D). This is unexpected, because the larvae used in this analysis (with 3–4 gill slits) possess only around 20 pairs of somites (Schubert et al., 2001), and amphioxus PGCs are deposited near the forming myomere boundaries only after the mid-neurula stage (with 8 pairs of somite) (Zhang et al., 2013). Moreover, the amputated posterior end includes the whole tail bud and the PGCs within it. We expected the amputated animals to develop around 20-8 = 12 pairs of gonads under the 8th–20th myomeres. To explain this paradox, we suggest that the amputated larvae may have also regenerated PGCs during recovery of the posterior end.

### The Posterior End of 4-Gill-Slits F0 Larvae Carries Similar Mutation Efficacy and Type to That in Their Tail Tips After Metamorphosis

**FIGURE 5:**
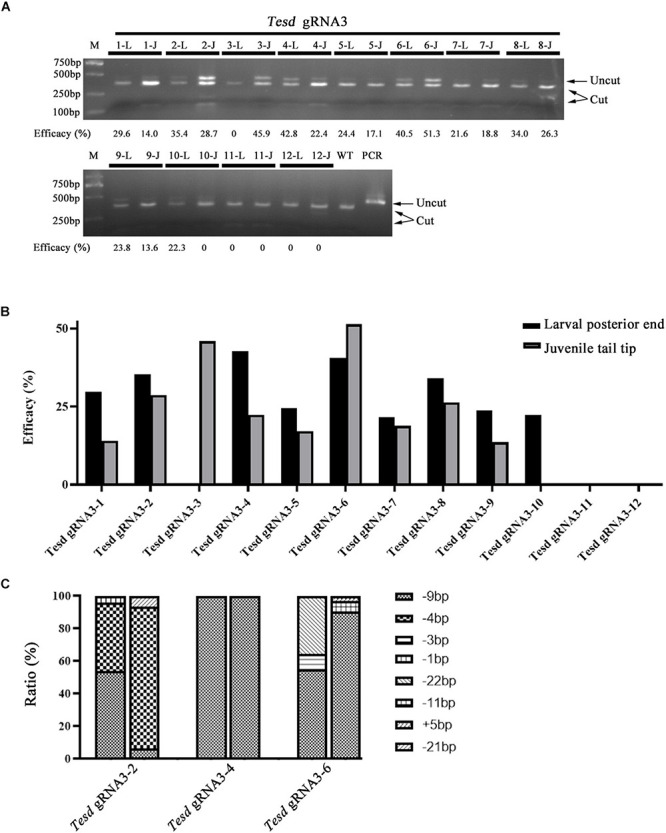
Mutation efficacy and type detected in posterior ends of 4-gill-slits larvae and their tail tips after metamorphosis. **(A)** Mutation efficacy detection by restriction enzyme assay. Twelve F0 individuals injected with Cas9 protein and *Tesd* gRNA3 were examined. The induced mutation efficacies (estimated as percentages of uncut PCR products) are labeled under the gel image. L represents efficacy detected in the posterior end of each individual at the larval stage, and J represents efficacy detected in their tail tips at the juvenile stage. **(B)** Graphic view of the mutation efficacies shown in **A**. **(C)** Mutation types detected in the posterior ends and tail tips of three F0 individuals (#2, #4, and #6). For each individual, the first column represents mutation types detected in the posterior ends and the second column represents those detected in the tail tips. Each type of mutation is marked with a different symbol.

We further examined if F0 individuals injected with Cas9 protein and gRNAs carry similar mutation efficacies and types in the posterior end at 4-gill-slits stage and tail tips at early juvenile stage. Twelve individuals injected with the *TesD* gRNA were used in the analysis. Among them, ten showed similar mutation efficacies among the two different tissues (*R*^2^ = 0.6871), while the other two carried mutations only in the posterior end at 4-gill-slits stage (#10) or only in the tail tip at early juvenile stage (#3) (Figures 5A,B). Further genotyping analysis of 3 individuals (#2, #4, and #6) revealed that they all carried similar major mutation types between the posterior ends at 4-gill-slits stage and tail tips at early juvenile stage (*R*^2^ = 0.5834) (Figure 5C and Supplementary Figure 3A).

## Conclusion

In summary, we here show that the mutation efficacy and type, and the presence (or absence) of transgene in F0 amphioxus gametes are strongly correlated with and can be reliably predicted by that in their tail tips. This is probably due to the special PGCs distribution mode in this group of animals. To our knowledge, such correlations have not been reported in other animals, including regularly used model organisms such as zebrafish and mouse. Our result also demonstrates that amphioxus larvae with 3–4 gill slits have strong posterior end regeneration potential and are able to fully recover after amputation within 6 days. Moreover, the amputated larvae can survive to adulthood at very high ratios and develop a normal number of gonads. We also show that F0 animals carry similar mutation efficacies and types in their posterior end at 4-gill-slits stage and their tail tips at early juvenile stage. These results, together with the method recently developed for genotyping amphioxus embryos and larvae with tiny piece of tissues (Holland and Li, 2021), suggest a great potential for obtaining F0 animals with desired mutations and transgenes at as early as the 3 gill-slit larval stage. We believe this will greatly accelerate, simplify and reduce the cost of amphioxus transgenic line establishment.

## Data Availability Statement

The original contributions presented in the study are included in the article/Supplementary Material, further inquiries can be directed to the corresponding author/s.

## Author Contributions

GL designed the study and conceived the experiments. JZ, XW, and GL performed the experiments, analyzed the data, compiled the figures, wrote, and revised the manuscript. XW and GL generated Invs and Cyp19a2 mutants. YZ generated Wnt3 and VegT mutants. LS and GL generated Mop mutants. LZ and QY generated XlSlug(S)-mCherry and BfPou4-mCherry transgenic lines, respectively. CS and GL performed larvae regeneration experiment. All authors have read and approved the final version of the manuscript.

## Conflict of Interest

The authors declare that the research was conducted in the absence of any commercial or financial relationships that could be construed as a potential conflict of interest.

## Publisher’s Note

All claims expressed in this article are solely those of the authors and do not necessarily represent those of their affiliated organizations, or those of the publisher, the editors and the reviewers. Any product that may be evaluated in this article, or claim that may be made by its manufacturer, is not guaranteed or endorsed by the publisher.

## Supplementary Material

Supplementary Figure 1 Correlation between mutation efficacy in tail tips and gametes of F0 individuals. **(A–C)** Mutation detection in tail tips (T) and gametes (G) of F0 individuals injected with *Wnt3* gRNA3 **(A)**, *Cyp19a2* gRNA2 **(B)** and *Cyp19a2* gRNA1 **(C)**. The induced mutation efficacies (estimated as percentages of uncut PCR products) are labeled under the gel image.

Supplementary Figure 2 Correlation between mutation type in tail tips and gametes of F0 individuals. **(A–J)** Mutation types detected in tail tips and gametes of two F0 females (♀1,♀3) injected with *Invs* gRNA1 **(A,B)**, two F0 females (♀2,♀3) injected with *Wnt3* gRNA3 **(C,D)**, one F0 male (♂1) injected with *VegT* gRNA4 **(E)**, two F0 females (♀1,♀2) injected with *Cyp19a2* gRNA2 (F-G) and three F0 (♂7, ♀2, ♀3) injected with *Mop* gRNA1 **(H–J)**. The gRNA target site is underlined and the restriction enzyme recognition sequences is marked in italics.

Supplementary Figure 3 Regeneration of amphioxus larvae that have undergone posterior end amputation and their gonad development. Fourteen larvae with 3–4 gill slits were amputated and 11 of them survived to adulthood. **(A)** Nine of the amputated larvae (1–9) and three of the posterior ends (10–11) amputated. The scale bar (200 μm) on 1 also applies to panels 2–9, and that on 10 (100 μm) applies to panel 11 too. **(B)** Thirteen of the amputated larvae 3 days after amputation. The scale bar (2 mm) on 1 applies to all panels. **(C)** All 14 amputated larvae 6 days after amputation. The scale bar (2 mm) on 1 applies to all panels. **(D)** Left- and right-side gonads of the 11 survived animals. Right-side gonads of animal 11 are not shown, but they can be scored from the picture taken from its left side.

Supplementary Figure 4 Mutation types detected in posterior ends of three 4-gill-slit larvae injected with *Tesd* gRNA3 and tail tips of them after metamorphosis. The gRNA target site is underlined and the restriction enzyme recognition sequences is marked in italics. Numbers after ^∗^ represents colony numbers carrying each mutation type.

## References

[B1] Benito-Gutierrez E. Weber H. Bryant D. V. Arendt D. (2013). Methods for generating year-round access to amphioxus in the laboratory. *PloS One* 8:e71599 10.1371/journal.pone.0071599 23990962 PMC3753313

[B2] Bertrand S. Escriva H. (2011). Evolutionary crossroads in developmental biology: amphioxus. *Development* 138 4819–4830. 10.1242/dev.066720 22028023

[B3] Delsuc F. Brinkmann H. Chourrout D. Philippe H. (2006). Tunicates and not cephalochordates are the closest living relatives of vertebrates. *Nature* 439 965–968. 10.1038/nature04336 16495997

[B4] Escriva H. (2018). My favorite animal, amphioxus: unparalleled for studying early vertebrate evolution. *Bioessays* 40:1800130. 10.1002/bies.201800130 30328120

[B5] Holland L. Z. Li G. (2021). Laboratory culture and mutagenesis of amphioxus (*Branchiostoma floridae*). *Methods Mol. Biol.* 2219 1–29. 10.1007/978-1-0716-0974-3_1 33074531

[B6] Hu G. Li G. Wang H. Wang Y. (2017). Hedgehog participates in the establishment of left-right asymmetry during amphioxus development by controlling Cerberus expression. *Development* 144 4694–4703. 10.1242/dev.157172 29122841

[B7] Huang S. Chen Z. Yan X. Yu T. Huang G. Yan Q. (2014). Decelerated genome evolution in modern vertebrates revealed by analysis of multiple lancelet genomes. *Nat. Commun.* 5:5896 10.1038/ncomms6896 25523484 PMC4284660

[B8] Li G. Feng J. Lei Y. Wang J. Wang H. Shang L.-K. (2014a). Mutagenesis at specific genomic loci of amphioxus *Branchiostoma* belcheri Using TALEN Method. *J. Genet. Genom.* 41 215–219. 10.1016/j.jgg.2014.02.003 24780619 PMC4535448

[B9] Li G. Liu X. Xing C. Zhang H. Shimeld S. M. Wang Y. (2017). Cerberus-Nodal-Lefty-Pitx signaling cascade controls left-right asymmetry in amphioxus. *Proc. Natl. Acad. Sci. U.S.A.* 114 3684–3689. 10.1073/pnas.1620519114 28320954 PMC5389317

[B10] Li G. Shu Z. Wang Y. (2014b). Year-round reproduction and induced spawning of Chinese amphioxus, *Branchiostoma* belcheri, in laboratory. *PloS One* 9:e99264. 10.1371/journal.pone.0099264 PMC3784433 24086537

[B11] Li G. Xi Y. Shu Z. H. Chen X. Y. Wang Y. Q. (2012). Consecutive spawnings of Chinese amphioxus, *Branchiostoma* belcheri, in captivity. *PloS One* 7:e50838. 10.1371/journal.pone.0050838 23251392 PMC3520940

[B12] Liu X. Li G. Feng J. Yang X. Wang Y.-Q. (2013). An efficient microinjection method for unfertilized eggs of Asian amphioxus *Branchiostoma* belcheri. *Dev. Genes Evol.* 223 269–278. 10.1007/s00427-013-0441-0 23584404

[B13] Marletaz F. Firbas P. N. Maeso I. Tena J. J. Bogdanovic O. Perry M. (2018). Amphioxus functional genomics and the origins of vertebrate gene regulation. *Nature* 564 64–70. 10.1038/s41586-018-0734-6 30464347 PMC6292497

[B14] Poss S. G. Boschung H. T. (1996). Lancelets (*Cephalochordata: Branchiostomatidae*): how many species are valid? *Isr. J. Zool.* 42 13–66.

[B15] Putnam N. H. Butts T. Ferrier D. E. K. Furlong R. F. Hellsten U. Kawashima T. (2008). The amphioxus genome and the evolution of the chordate karyotype. *Nature* 453 1064–U1063. 10.1038/nature06967 18563158

[B16] Ren Q. Zhong Y. Huang X. Leung B. Xing C. Wang H. (2020). Step-wise evolution of neural patterning by Hedgehog signalling in chordates. *Nat. Ecol. Evol.* 4 1247–1255. 10.1038/s41559-020-1248-9 32661406

[B17] Schubert M. Holland L. Z. Stokes M. D. Holland N. D. (2001). Three amphioxus Wnt genes (AmphiWnt3. AmphiWnt5, and AmphiWnt6) associated with the tail bud: the evolution of somitogenesis in chordates. *Dev. Biol.* 240 262–273. 10.1006/dbio.2001.0460 11784062

[B18] Shi C. Huang J. Chen S. Li G. Wang Y. (2018). Generation of two transgenic amphioxus lines using the Tol2 transposon system. *J. Genet. Genom.* 45 513–516. 10.1016/j.jgg.2018.06.002 30268359

[B19] Somorjai I. M. L. Somorjai R. L. Garcia-Fernandez J. Escriva H. (2012). Vertebrate-like regeneration in the invertebrate chordate amphioxus. *Proc. Natl. Acad. Sci.U.S.A.* 109 517–522. 10.1073/pnas.1100045109 22203957 PMC3258630

[B20] Su L. Shi C. Huang X. Wang Y. Li G. (2020). Application of CRISPR/Cas9 nuclease in amphioxus genome editing. *Genes* 11:1311. 10.3390/genes11111311 33167309 PMC7694359

[B21] Vallin J. Thuret R. Giacomello E. Faraldo M. M. Broders F. (2001). Cloning and characterization of threexenopus slug promoters reveal direct regulation by Lef/β-catenin signaling. *J. Biol. Chem.* 276 30350–30358. 11402039 10.1074/jbc.M103167200

[B22] Zhang Q.-J. Luo Y.-J. Wu H.-R. Chen Y.-T. Yu J.-K. (2013). Expression of germline markers in three species of amphioxus supports a preformation mechanism of germ cell development in *Cephalochordates*. *Evodevo* 4:17. 10.1186/2041-9139-4-17 23777831 PMC3735472

[B23] Zhang Q.-J. Sun Y. Zhong J. Li G. Lue X.-M. Wang Y.-Q. (2007). Continuous culture of two lancelets and production of the second filial generations in the laboratory. *J. Exp. Zool. B Mol. Dev. Evol.* 308B 464–472. 10.1002/jez.b.21172 17497691

[B24] Zhong Y. Herrera-Ubeda C. Garcia-Fernandez J. Li G. Holland P. W. H. (2020). Mutation of amphioxus Pdx and Cdx demonstrates conserved roles for ParaHox genes in gut, anus and tail patterning. *BMC Biol.* 18:68 10.1186/s12915-020-00796-2 32546156 PMC7296684

[B25] Zhu X. Shi C. Zhong Y. Liu X. Yan Q. Wu X. (2020). Cilia-driven asymmetric Hedgehog signalling determines the amphioxus left-right axis by controlling Dand5 expression. *Development* 147:dev182469. 10.1242/dev.182469 31826864

