# Supplementary figures and images for: A Potential Method for Rapid Screening of Amphioxus Founder Harboring Germline Mutation and Transgene
Source: Front Cell Dev Biol. 2021 Aug 12;9:702290. doi: 10.3389/fcell.2021.702290 (PMC8387717; doi:10.3389/fcell.2021.702290)

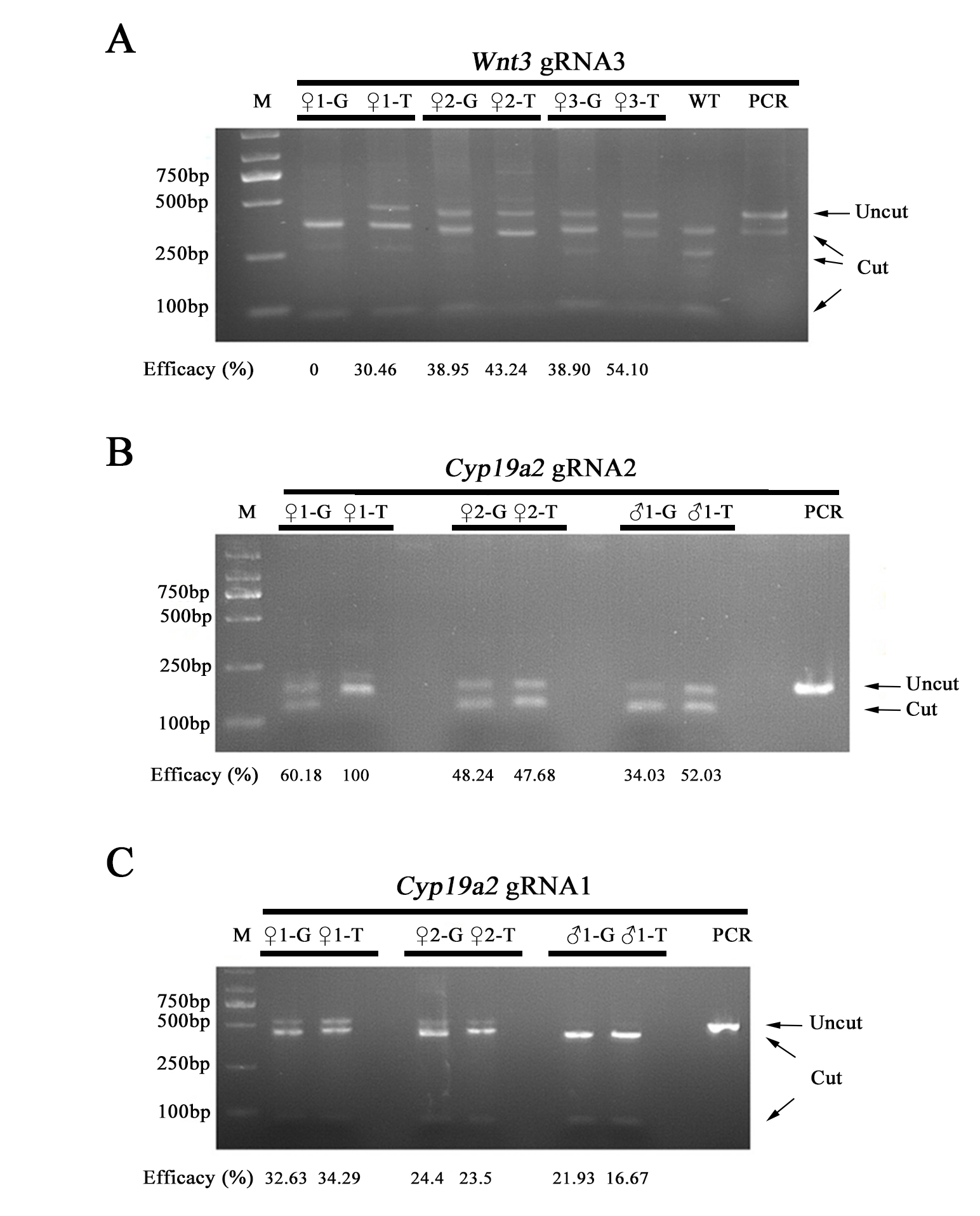

Supplement: Supplementary Figure 1 — Correlation between mutation efficacy in tail tips and gametes of F0 individuals. (A–C) Mutation detection in tail tips (T) and gametes (G) of F0 individuals injected with Wnt3 gRNA3 (A), Cyp19a2 gRNA2 (B) and Cyp19a2 gRNA1 (C). The induced mutation efficacies (estimated as percentages of uncut PCR products) are labeled under the gel image. [file Image_1.TIF]

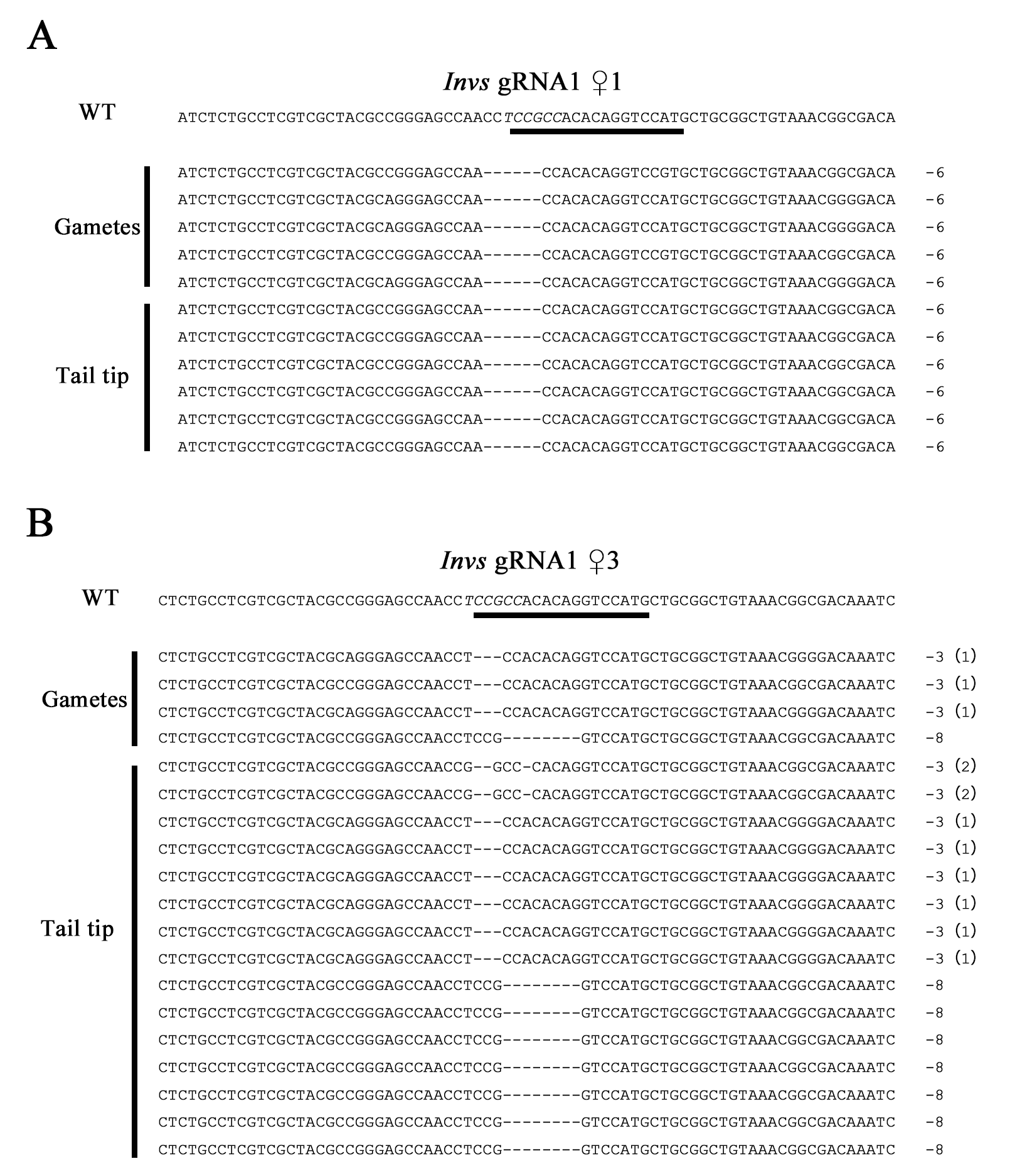

Supplement: Supplementary Figure 2 — Correlation between mutation type in tail tips and gametes of F0 individuals. (A–J) Mutation types detected in tail tips and gametes of two F0 females (♀1,♀3) injected with Invs gRNA1 (A,B), two F0 females (♀2,♀3) injected with Wnt3 gRNA3 (C,D), one F0 male (♂1) injected with VegT gRNA4 (E), two F0 females (♀1,♀2) injected with Cyp19a2 gRNA2 (F-G) and three F0 (♂7, ♀2, ♀3) injected with Mop gRNA1 (H–J). The gRNA target site is underlined and the restriction enzyme recognition sequences is marked in italics. [file Image_2.TIF]

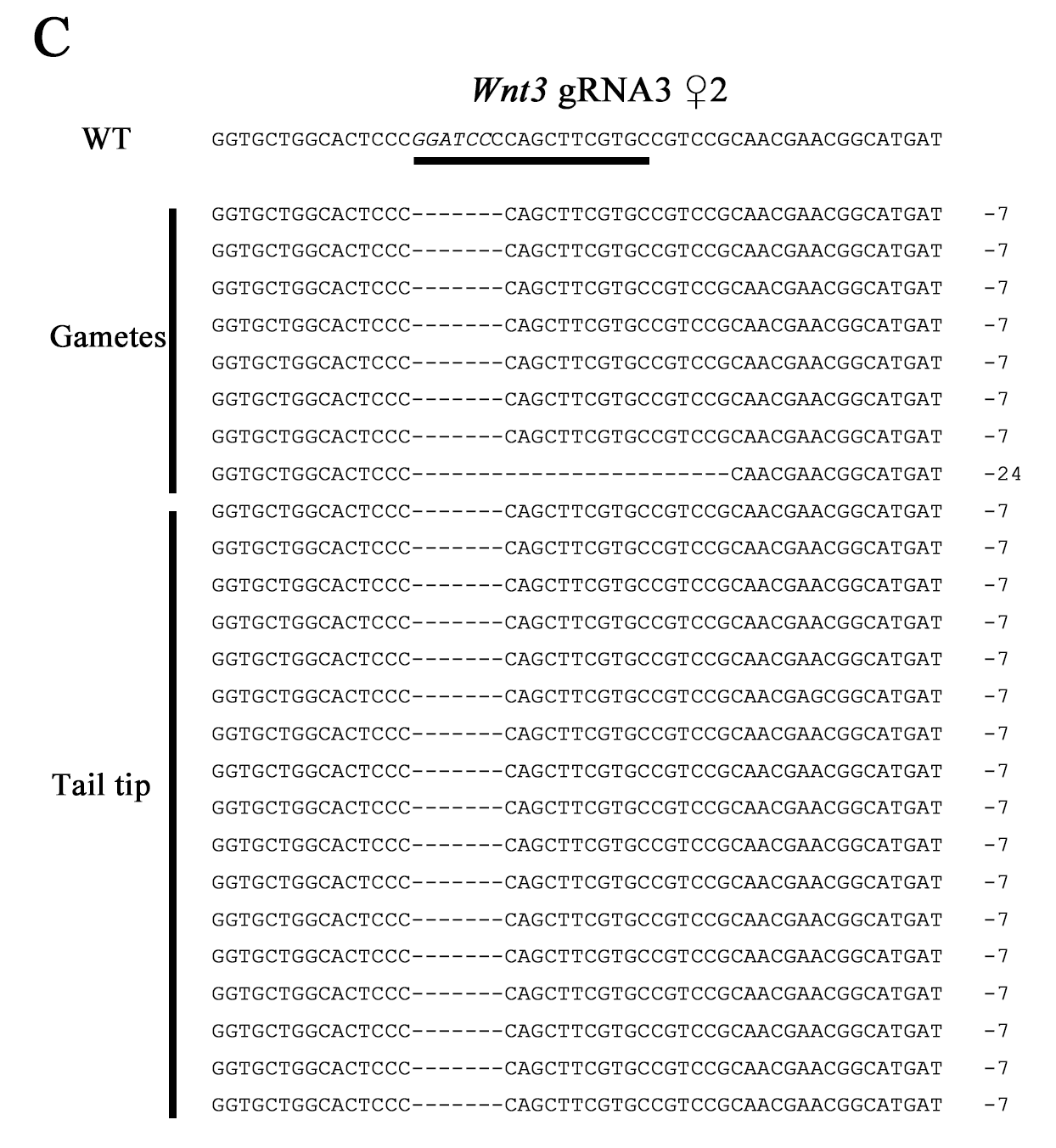

Supplement: Supplementary Figure 3 — Regeneration of amphioxus larvae that have undergone posterior end amputation and their gonad development. Fourteen larvae with 3–4 gill slits were amputated and 11 of them survived to adulthood. (A) Nine of the amputated larvae (1–9) and three of the posterior ends (10–11) amputated. The scale bar (200 μm) on 1 also applies to panels 2–9, and that on 10 (100 μm) applies to panel 11 too. (B) Thirteen of the amputated larvae 3 days after amputation. The scale bar (2 mm) on 1 applies to all panels. (C) All 14 amputated larvae 6 days after amputation. The scale bar (2 mm) on 1 applies to all panels. (D) Left- and right-side gonads of the 11 survived animals. Right-side gonads of animal 11 are not shown, but they can be scored from the picture taken from its left side. [file Image_3.TIF]

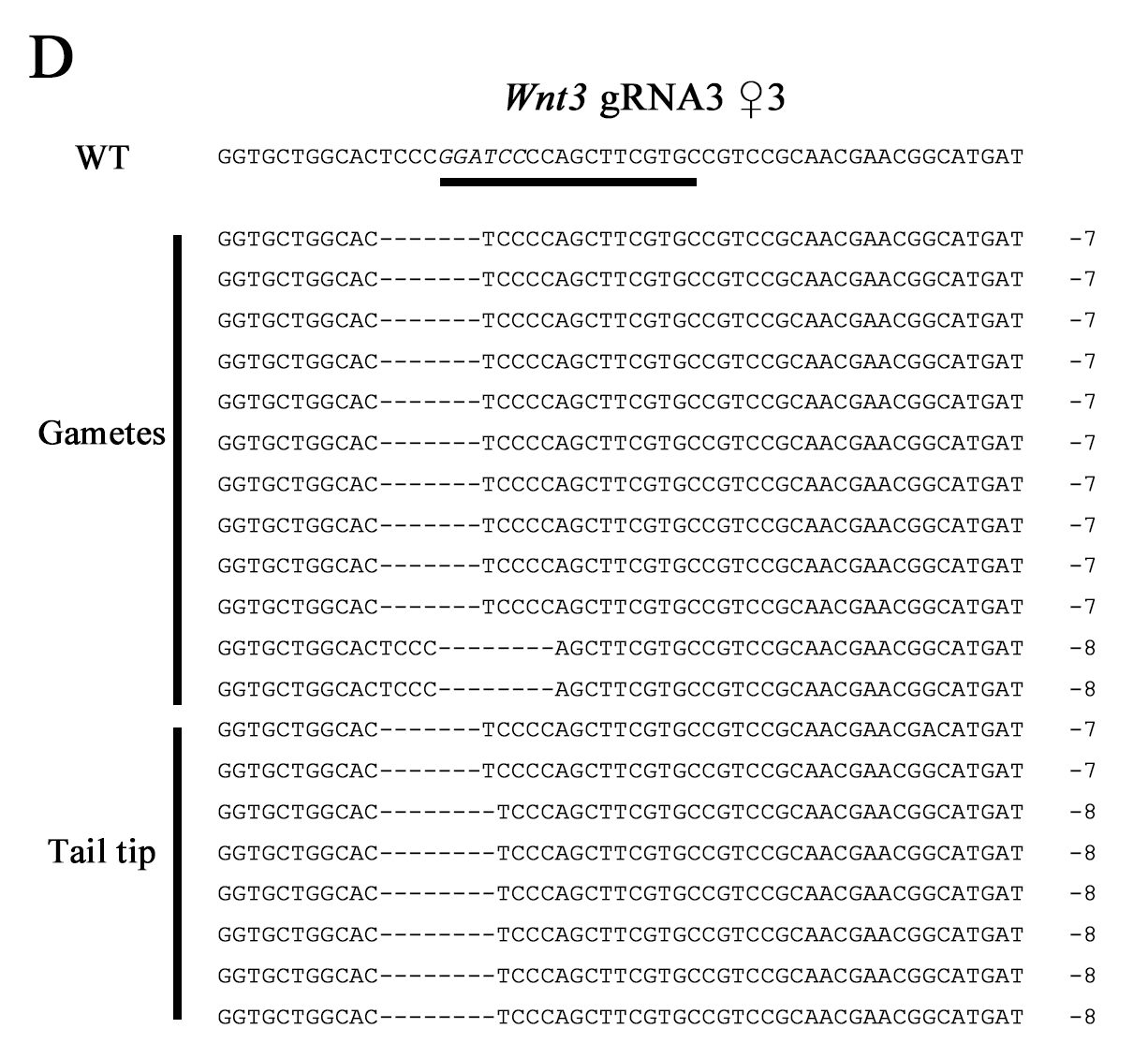

Supplement: Supplementary Figure 4 — Mutation types detected in posterior ends of three 4-gill-slit larvae injected with Tesd gRNA3 and tail tips of them after metamorphosis. The gRNA target site is underlined and the restriction enzyme recognition sequences is marked in italics. Numbers after ∗ represents colony numbers carrying each mutation type. [file Image_4.TIF]

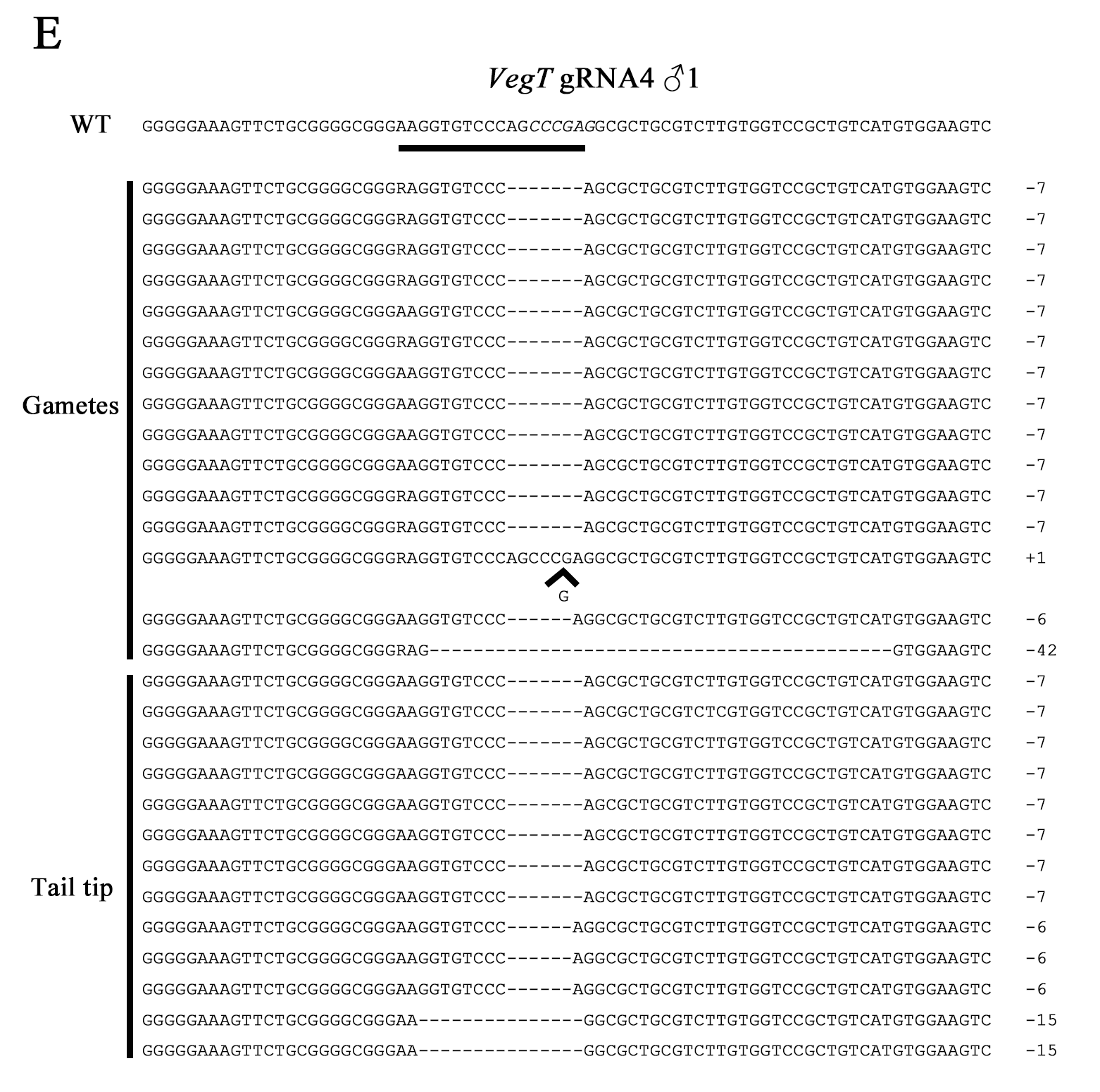

Supplement: Supplementary file 5 [file Image_5.TIF]

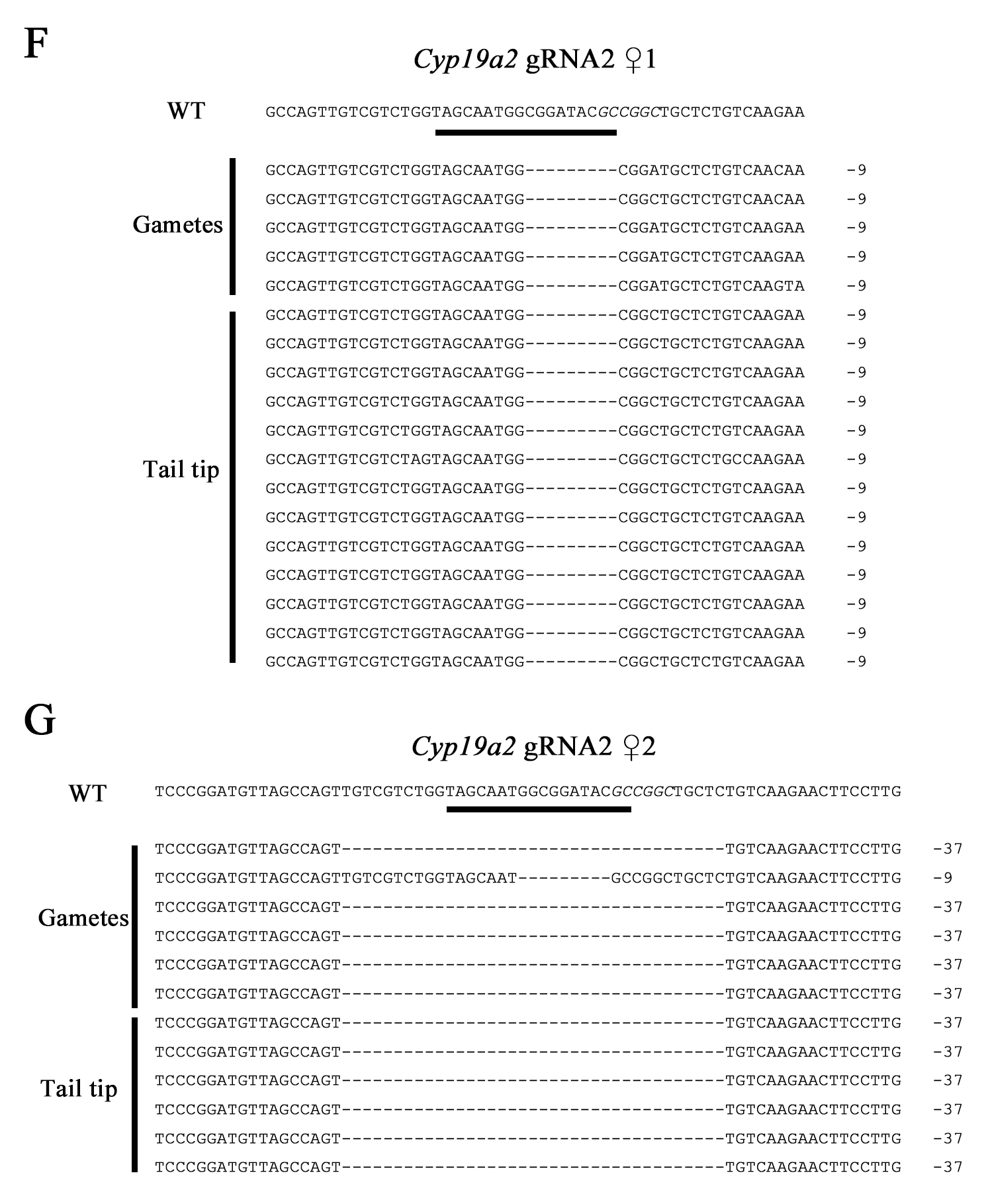

Supplement: Supplementary file 6 [file Image_6.TIF]

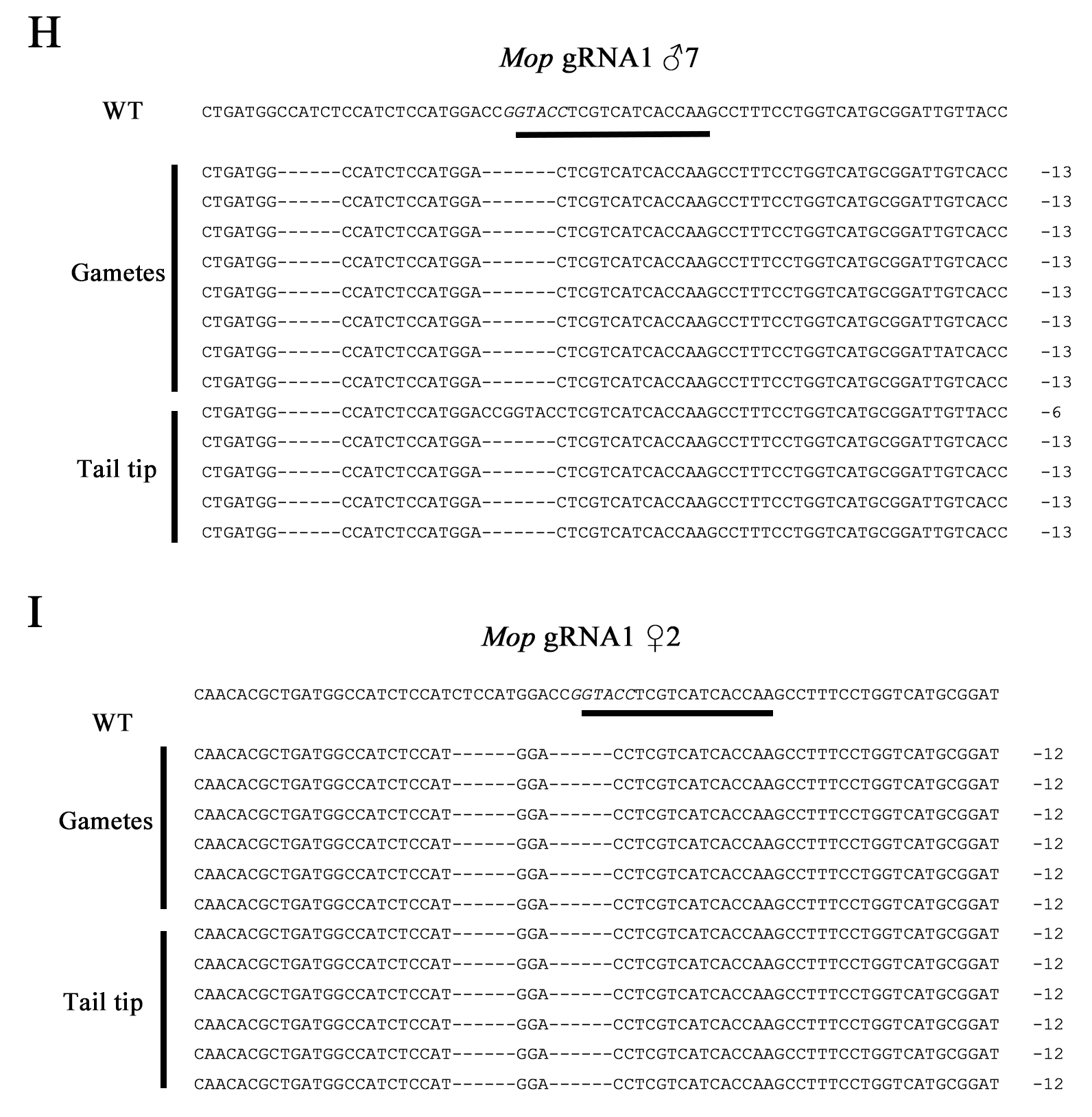

Supplement: Supplementary file 7 [file Image_7.TIF]

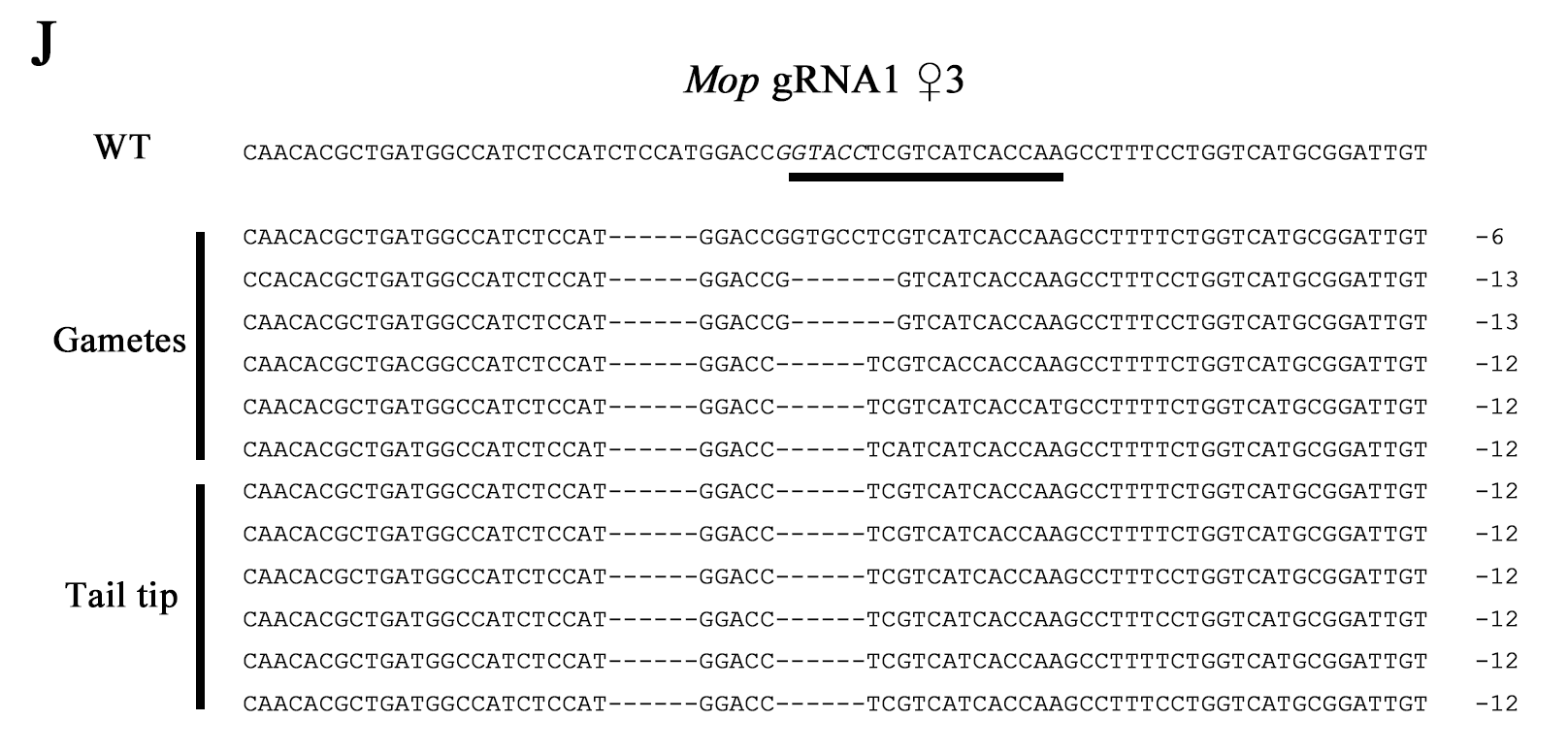

Supplement: Supplementary file 8 [file Image_8.TIF]

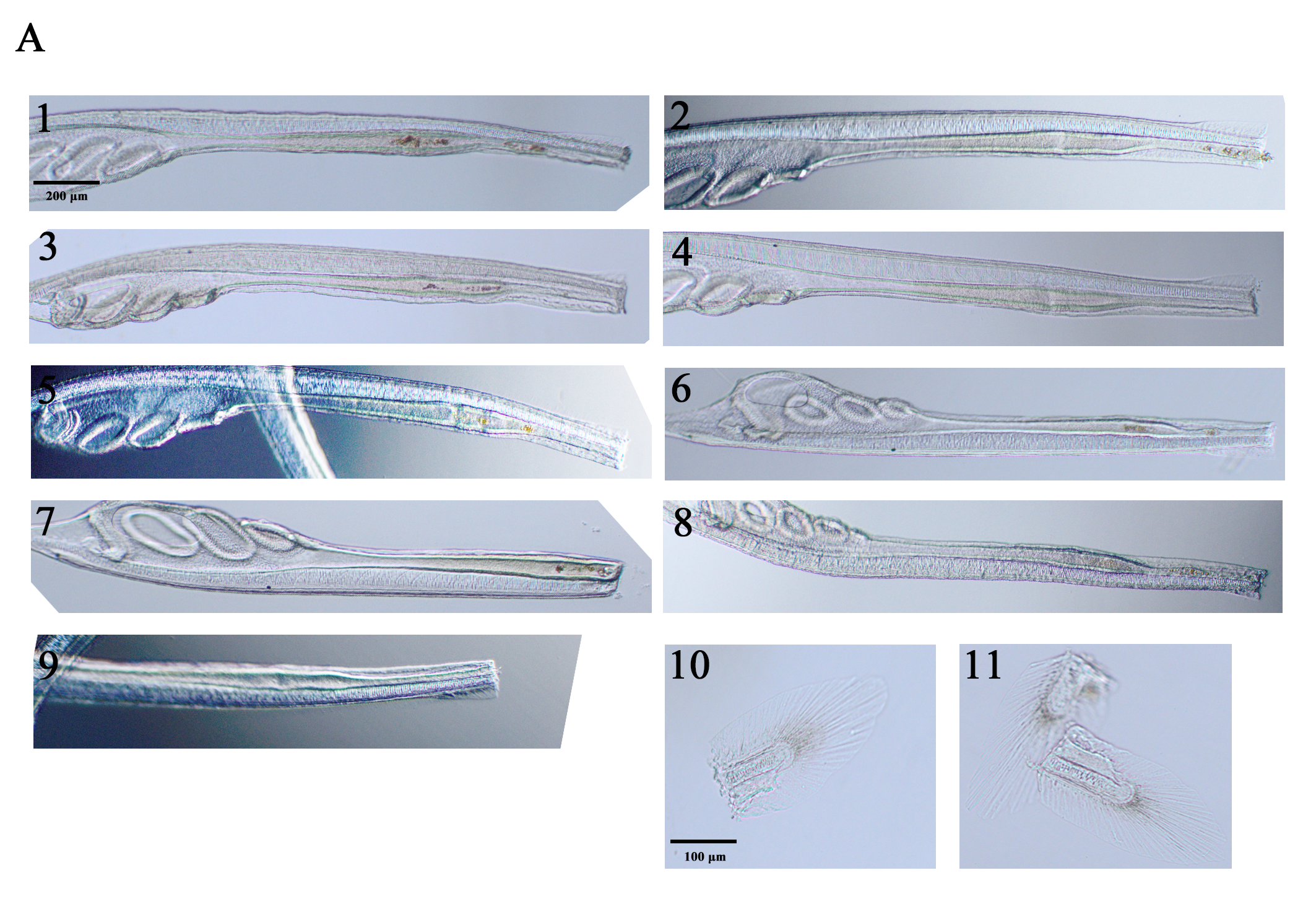

Supplement: Supplementary file 9 [file Image_9.TIF]

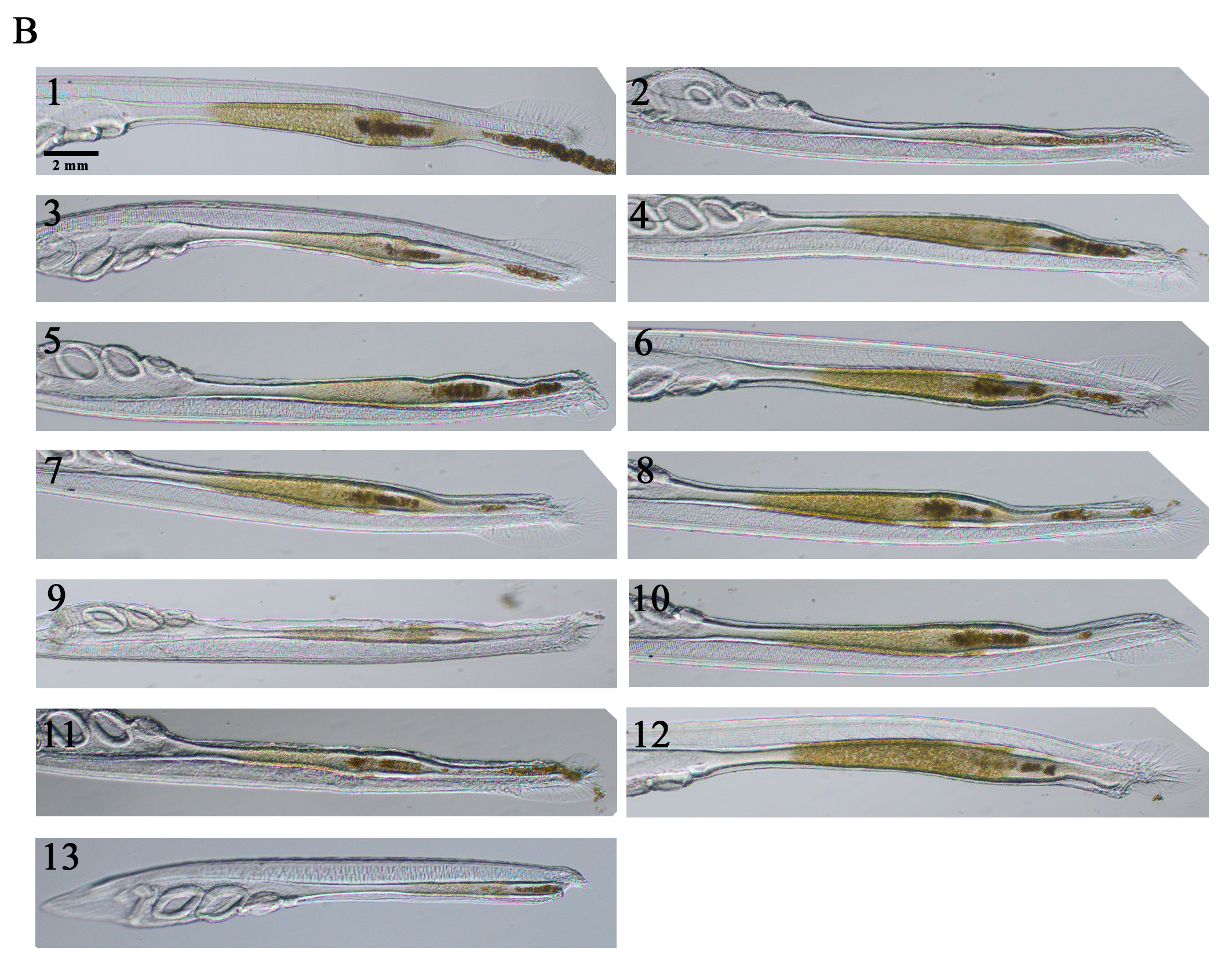

Supplement: Supplementary file 10 [file Image_10.TIF]

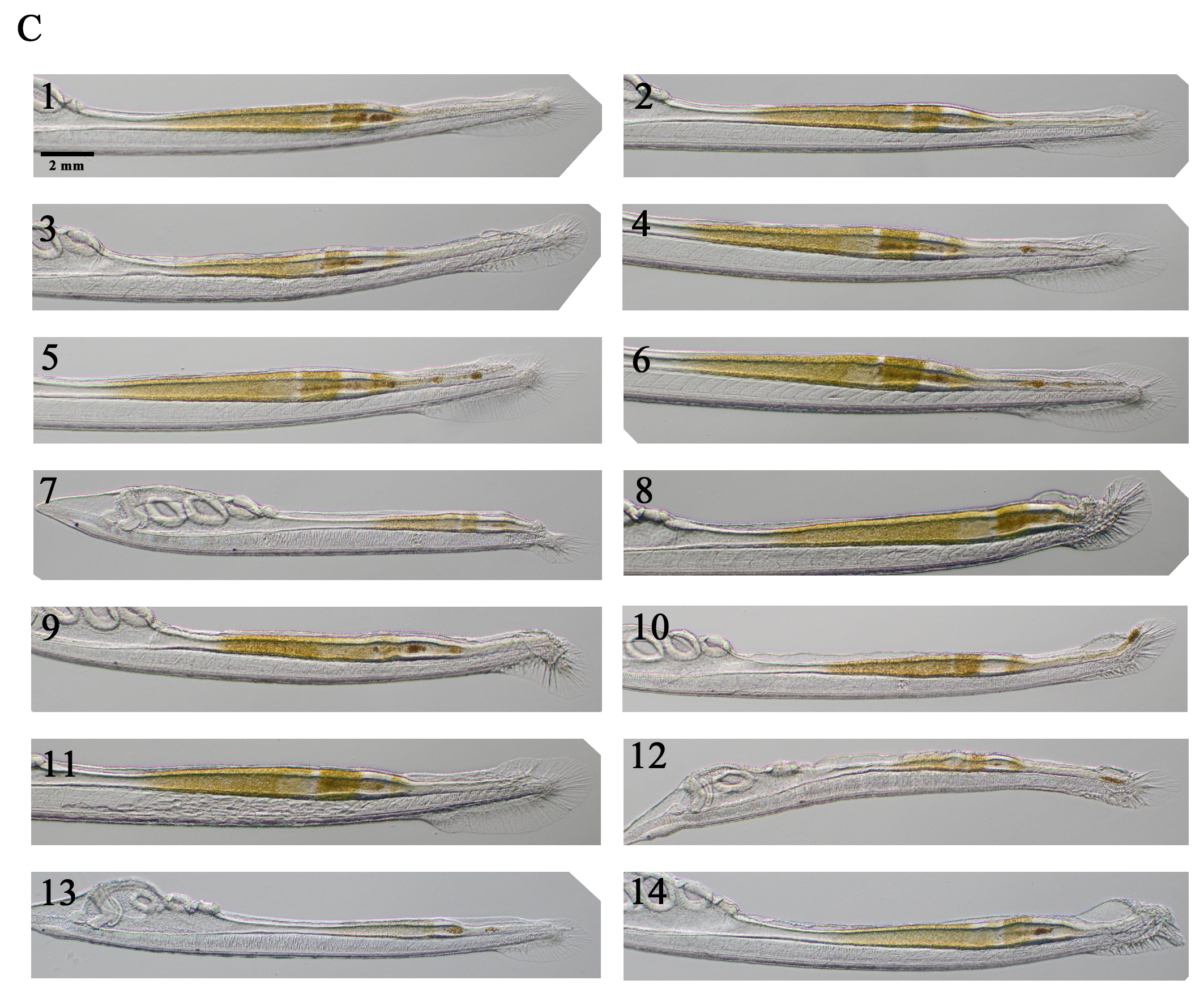

Supplement: Supplementary file 11 [file Image_11.TIF]

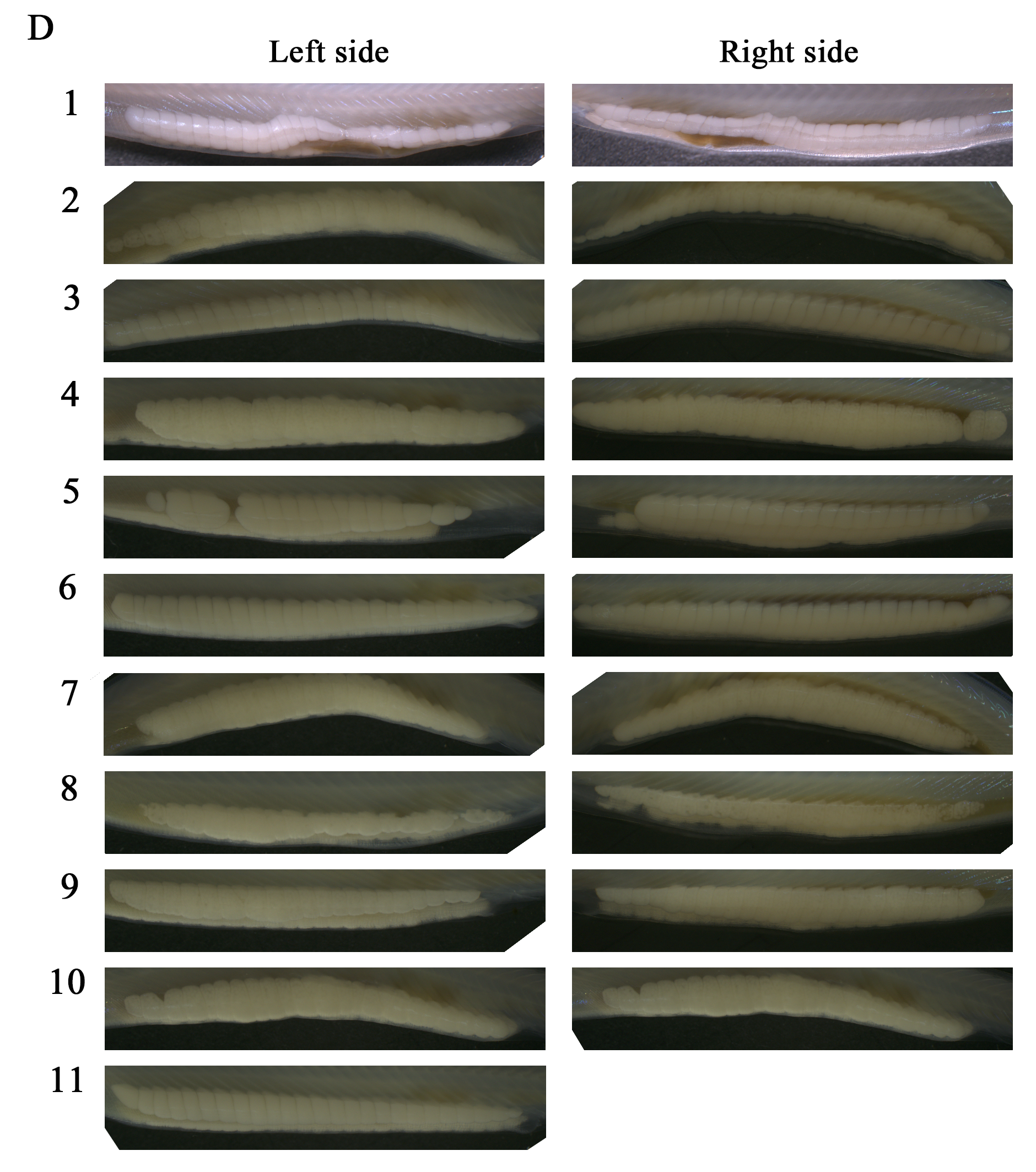

Supplement: Supplementary file 12 [file Image_12.TIF]

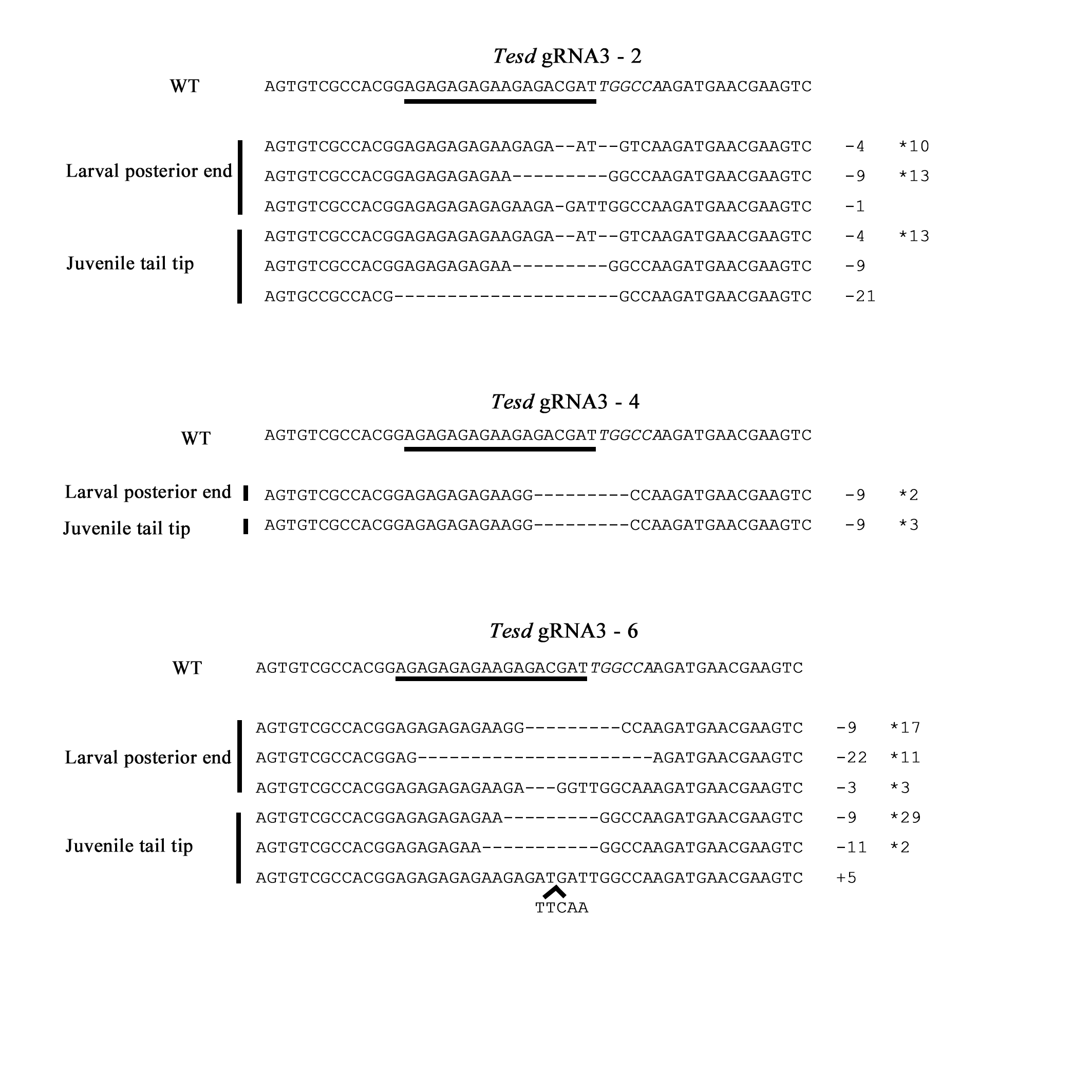

Supplement: Supplementary file 13 [file Image_13.TIF]
